# Risk Stratification for Sudden Cardiac Death: Current Approaches and Predictive Value

**DOI:** 10.2174/157340309787048130

**Published:** 2009-01

**Authors:** Gustavo Lopera, Anne B. Curtis

**Affiliations:** 1 Division of Cardiology, University of Miami/Miller School of Medicine, Miami, FL, USA; 2 Division of Cardiology, University of South Florida, Tampa, FL. USA

**Keywords:** Sudden cardiac death, risk stratification markers.

## Abstract

Sudden cardiac death (SCD) is a serious public health problem; the annual incidence of out-of-hospital cardiac arrest in North America is approximately 166,200. Identifying patients at risk is a difficult proposition. At the present time, left ventricular ejection fraction (LVEF) remains the single most important marker for risk stratification. According to current guidelines, most patients with LVEF <35% could benefit from prophylactic ICD implantation, particularly in the setting of symptomatic heart failure. Current risk stratification strategies fail to identify patients at risk of SCD in larger population groups encompassing a greater number of potential SCD victims. However, the best approach to identifying patients and the value of various risk stratification tools is not entirely clear. The goal of this review is to discuss the problem of SCD and the value of the different risk stratification markers and their potential clinical use either alone or in combination with other risk stratification markers.

## INTRODUCTION

Sudden cardiac death (SCD) is a serious public health problem, yet identifying patients at risk is a difficult proposition. Implantable cardioverter defibrillators (ICDs) have been shown to be beneficial in improving survival in patients at risk for SCD. However, the best approach to identifying patients and the value of various risk stratification tools is not entirely clear. The goal of this review is to discuss the problem of SCD in the context of methods to identify patients at risk, their relative value, and the concepts of relative and absolute risk reduction and number needed to treat as they help to frame the discussion of risk stratification and utilization of ICD therapy. 

## STATEMENT OF THE PROBLEM: POPULATIONS AT RISK, RISK REDUCTION, AND NUMBER NEEDED TO TREAT

SCD is generally defined as a natural unexpected death due to cardiac causes occurring within one hour of the onset of acute symptoms [1]. Although difficult to measure, the annual incidence of out-of-hospital cardiac arrest in North America is approximately 0.55 per 1000 population; this implies that approximately 166,200 out-of-hospital cardiac arrests occur annually [2]. 

**Fig. (1):**
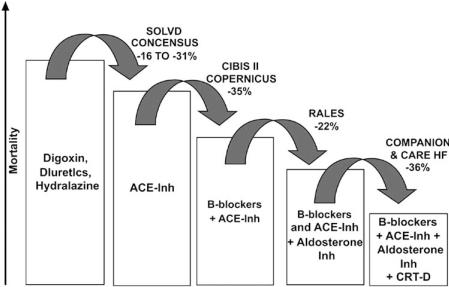
Results from clinical trials showing incremental decreases in mortality when added onto previous therapy. (Reprinted with permission Elsevier Limited).

Primary prevention trials of ICD therapy have established depressed left ventricular ejection fraction (LVEF) as the single most important risk stratification tool to identify individuals with a high risk of SCD [3-5]. In these trials, ICD therapy has provided an additional one-third incremental protection against all-cause mortality and SCD to the benefit obtained with optimal heart failure therapy alone [6] (Fig. **1**). 

Despite the proven benefit of ICD therapy, risk stratification strategies based predominantly on severely depressed LVEF may not identify up to two-thirds of SCD victims who could potentially benefit from prophylactic ICDs and/or other therapeutic interventions [7-10]. This statistic is a natural consequence of the fact that the majority of potential SCD victims come from larger populations groups, such as those with coronary risk factors or the general population who either have preserved LVEFs or unrecognized LV dysfunction [11]. The implementation of prophylactic ICD therapy and/or other therapeutic intervention in these larger population groups is, at least partially, limited by their cost, risk-benefit ratio (number need to treat), impact on quality of life, the lack of reliable risk stratification tools in larger population groups, and lack of scientific evidence that ICD therapy improves survival in these lower risk populations. 

Risk stratification strategies to identified potential SCD victims have been extensively studied in the last three decades. Three distinct types of SCD risk stratifiers can be identified:

1. Markers of abnormal substrate or structural heart disease: LVEF, non-sustained ventricular tachycardia (NSVT), frequent ventricular ectopy, QRS duration;
2. Markers of abnormal repolarization or electrical instability: electrophysiologic studies (EPS), T wave alternans (TWA), signal averaged ECG (SAECG), QT dispersion;
3. Markers of abnormal autonomic balance: heart rate variability (HRV) and baroreceptor sensitivity (BRS).

At present, it is unclear if these risk markers identify the same patient populations or provide complementary risk stratification information. The widespread utilization of each of these risk markers in clinical practice depends on their power as predictors of major cardiovascular events in specific patient populations. 

Incorporation of a risk marker into clinical practice will depend on its power to predict adverse clinical outcomes if positive (positive predictive value, PPV) and/or their power to predict lack of adverse outcomes if negative (negative predictive value, NPV). Utilization of these markers also depends on the impact of their risk stratification power. For example, in the MADIT II trial, using LVEF ≤30% as a single risk stratifying tool, prophylactic ICD therapy reduced mortality from 19.8% in the conventional-therapy group to 14.2% in the defibrillator group during an average follow-up of 20 months. The relative risk reduction (RRR) of mortality was 31% and the absolute risk reduction (ARR) of mortality was 5.6%. That is, 18 defibrillators would need to be implanted to save a life (number needed to be treated, NNT) in MADIT II like patients [12,13]. The bottom line is that RRR and ARR are different ways of conveying the same information, yet they may be perceived very differently by patients and even by some health care providers. 

The practice of medicine is largely based on application of a therapy or intervention in many individuals to prevent or avoid an adverse outcome in some. For example, data from 16 individual trials of subjects treated with HMG-CoA reductase inhibitors (statin drugs) demonstrated a 28% RRR in cardiovascular mortality, but only a 1.7% decrease in ARR of cardiovascular mortality, during an average follow-up of 3.3 years. In the GISSI trial, thrombolytic therapy was associated with a RRR in mortality of 18%, but the ARR in mortality was 2.3% [11,14,15]. Yet, both these therapies are well accepted in the care of patients with cardiovascular disease with lesser effects on mortality than most of the ICD trials. 

## MARKERS OF ABNORMAL SUBSTRATE OR STRUCTURAL HEART DISEASE

### Depressed Left Ventricular Ejection Fraction

Primary prevention ICD trials had established depressed LVEF as the single most important risk stratification tool to identify individuals with a high risk of SCD. Data from seven primary prevention ICD trials have shown an average 28% RRR and 3% ARR of death in ICD-treated patients as compared to medical therapy [3-5]. However, this benefit is most apparent in patients with the lowest LVEFs. A mortality analysis from the primary prevention ICD trials showed a RRR of death of 29% in patients with LVEF ≤ 30%, but no significant mortality benefit for those with an LVEF >30% [4]. Similarly, in the AVID trial, patients with LVEF <35% had a significant survival benefit, whereas in patients with an LVEF ≥35, there was no difference in survival between ICD-treated patients and the medical therapy group. 

Nonetheless, LVEF alone will not adequately identify populations at risk of SCD. In the Maastricht circulatory arrest registry, 56.5% of the SCD victims had an LVEF >30% and 20% had a LVEF >50%. Moreover, the interval between the last myocardial infarction (MI) and SCD was >2 years in 66% of the victims (mean 6.5 years), and the interval between the first presentation with heart failure and SCD was 4.3±6.3 years, bringing into question the concept that cardiac events are time-dependent risk factors for SCD [8-11]. 

Similarly, in the Oregon Sudden Unexpected Death Study, the LVEF was severely (<35%) reduced in 36 patients (30%), mildly to moderately (36-54% LVEF) reduced in 27 patients (22%), and normal in 58 patients (48%). Those with normal LVEF were younger and had a lower prevalence of established coronary artery disease [9]. 

The mean LVEF in the ICD trials for secondary prevention of SCD was 32% (AVID), 34% (CIDS) and 45% (CASH). Consequently, most patients in these trials had an LVEF >30% [16-18]. In these trials, an average of 35% RRR and 7.5% ARR of death in ICD-treated patients as compared to medical therapy was observed, over a follow-up period of 11 to 36 months [3-5]. 

The prognostic value of a depressed LVEF appears to be influenced by other factors. Analysis of data from 674 patients enrolled in the MUSTT study who did not receive antiarrhythmic or ICD therapy revealed that other variables, such as: functional class, history of heart failure, NSVT, age, left ventricular conduction abnormalities, inducible sustained ventricular tachycardia, and atrial fibrillation influence arrhythmic death and total mortality risk. Patients with an EF ≤30% but no other risk factors had a low predicted mortality risk. Patients with EF >30% and other risk factors may have a higher mortality and a higher risk of sudden death than some patients with EFs ≤30%. Thus, the risk of SCD in patients with coronary artery disease depends on multiple variables in addition to EF [19]. 

### Non-sustained Ventricular Tachycardia and Ventricular Ectopy

The frequency and complexity of ventricular arrhythmias increases as the LVEF deteriorates. However, there is dissociation between the frequency of ventricular arrhythmias and the prevalence of SCD among patients with heart failure. Thus, a higher proportion of patients with New York Heart Association functional class I-II die suddenly (50-60%) compared to patients with functional class III-IV (<30%), suggesting that NSVT and frequent ventricular ectopy are markers of abnormal substrate or structural heart disease rather than arrhythmic markers per se [20, 21]. This discordance between the prevalence of ventricular arrhythmias and outcomes was also corroborated in the CAST trial, which reported a significant increment in arrhythmic and non-arrhythmic cardiac death among post-MI patients treated with encainide and flecainide compared to placebo, despite suppression of ventricular ectopy with these drugs [22]. 

In a single center, prospective study of 700 post MI patients on adequate beta blocker therapy (>95% compliance) Huikuri *et al. *reported that SCD was weakly predicted by reduced EF (<0.40; p < 0.05), NSVT (p < 0.05), and an abnormal SAECG (p < 0.05), but not by autonomic markers (HVR, BRS) or standard ECG variables (QT dispersion, QT duration). The PPV of low EF, NSVT, and abnormal SAECG in predicting SCD (8%, 12%, and 13%, respectively) was lower than for non-sudden cardiac death (15%, 15%, and 18%, respectively) [23]. These observations indicate that low EF and NSVT are markers of increased cardiac mortality (arrhythmic and non-arrhythmic), but not good indicators of the specific mode of death. 

The role of NSVT as a risk stratification tool was evaluated in three of the major primary prevention ICD trials (MUSTT, MADIT, DEFINITE). In two of these trials (MADIT, MUSTT), NSVT combined with depressed LVEF and inducible VT at EPS identified patients that benefited from a prophylactic ICD. DEFINITE was a primary prevention trial in patients with dilated, non-ischemic cardiomyopathy. Although a history of NSVT was necessary for enrollment in the study, EPS have not been shown to be helpful in such patients and were not included in the study procedures. Altogether, these trials showed a RRR of the primary endpoint (arrhythmic death or cardiac arrest, MUSTT) or mortality (MADIT, DEFINITE) of 76%, 56% and 35%, respectively with ICD therapy [5]. 

The role of NSVT and frequent ventricular ectopy as risk stratifying tools in patients with LVEF >40% is not clear at the present time. 

### QRS Duration

A meta-analysis of primary prevention ICD trials showed that patients with QRS durations ≥120ms had a greater survival benefit than patients with QRS <120ms (RRR in mortality of 30% *vs.* 18%). However, the mortality benefit was significant in both groups [4]. 

In a prospective clinical trial, Chow *et al. *reported the prognostic value of TWA and QRS duration in 768 patients with ischemic cardiomyopathy (LVEF ≤35%) and no history of ventricular arrhythmias. During a mean follow-up of 18±10 months, 99 patients died. After multivariable adjustment, a non-negative (positive or indeterminate) TWA test result was associated with a significantly higher risk for all-cause mortality in patients without ICDs and for all-cause mortality and appropriate ICD shocks in patients with an ICD. A QRS >120 ms was not associated with mortality outcomes, regardless of whether a QRS duration >120, >140, or >160 ms was used [24]. 

Similarly, Bloomfield *et al. *reported the prognostic value of TWA and QRS duration in 177 MADIT II-like patients. During an average follow-up of 20+6 months, 20 patients died. The mortality rate was substantially lower among patients with a normal TWA (<9%) than the mortality rate in patients with a narrow QRS (<18.5%) [25]. 

These data suggest that QRS duration does not provide significant additional prognostic value over and above that of LVEF, NSVT and TWA. It is not clear if QRS duration could be used as a risk stratification tool when combined with other risk markers in different patient populations. 

## MARKERS OF ABNORMAL REPOLARIZATION OR ELECTRICAL INSTABILITY

### T Wave Alternans

Electrical alternans and, hence, action potential alternans result from changes in cardiac repolarization that occurs on a beat-to-beat basis. TWA testing involves measuring variations in the T wave morphology on an every-other-beat basis. Typically, this variation is on the order of a few microvolts and requires specialized signal processing methods. Two types of TWA testing are available: spectral analysis and modified moving average. Experimental and clinical studies have shown that abnormal TWA is associated with an increased risk of ventricular arrhythmias [26-28]. Most studies have found that the prognosis of patients with indeterminate TWA is similar to that of patients with abnormal or positive tests, so these patient groups are often combined. 

A meta-analysis of 19 prospective studies of TWA between 1990 and 2004, including 2,608 subjects with heart failure, ischemic and non-ischemic cardiomyopathies, post-MI, athletes, and healthy subjects reported an overall 19.3 % PPV of TWA for arrhythmic events, and a 97.2% NPV. Subjects with indeterminate TWA were excluded. There was no difference in predictive value between ischemic and nonischemic heart failure subgroups [29]. 

Results from early clinical trials using TWA as a risk stratification marker suggested that TWA has a similar prognostive value in ischemic and nonischemic cardiomyopathy, adds predictive value to the other risk markers (LVEF, EPS and NSVT), and identifies different populations at risk of arrhythmic events compared to those identified with LVEF, NSVT and EPS [30-33]. 

Bloomfield *et al. *evaluated the predictive value of TWA in a multicenter prospective trial of 549 patients with LVEF ≤ 40% (mean LVEF=25%) and no previous history of sustained ventricular arrhythmias. The primary end point was all-cause mortality or non-fatal sustained ventricular arrhythmias. During a mean follow-up of 20 ± 6 months, 51 endpoints occurred. The two-year event rate was 15.0% in the patients with a non-negative TWA and 2.5% in those with a negative TWA (hazard ratio (HR) = 6.5, p < 0.001). Interestingly, patients with a negative TWA and LVEF ≤ 30% had a lower two-year event rate (3.5%) than patients with a non-negative TWA test and an LVEF between 31% and 40% (11.8%), suggesting that a negative TWA can help to assess the predictive value of a markedly depressed LVEF and could improve assessment of the potential benefit of prophylactic ICD therapy [34]. 

In the ALPHA study, 446 patients with nonischemic cardiomyopathy and LVEF ≤40% were followed for 18 to 24 months. The primary endpoint was a combination of cardiac death and life-threatening arrhythmias. Primary endpoint rates in patients with abnormal and normal TWA tests were 9.9% (n=292) and 2.5% (n=154), respectively. Although the NPV of a negative TWA test at 12 and 18 months exceeded 97%, the PPV were relatively low (6.5% and 9.0% for the primary end point at 12 and 18 months) slightly increasing to 7.9% and 10.9% at 12 and 18 months, respectively, among patients with LVEF ≤35%. The authors concluded that patients with nonischemic cardiomyopathy, LVEF ≤40%, and negative TWA have a very good prognosis and are likely to benefit little from ICD therapy [35]. However, interpretation of these results and their usefulness in clinical practice is limited by the low incidence of primary endpoint events in both groups. It also brings into question of the clinical value of a test with an excellent NPV, but a low PPV. 

The preliminary results of the prospective clinical trial Alternans Before Cardioverter Defibrillator (ABCD) in 566 patients with coronary artery disease, an LVEF ≤40%, and a history of nonsustained VT but no sustained ventricular arrhythmias who underwent both TWA and EPS were presented at the 2006 AHA Annual Scientific Sessions (Heartwire/The Heart.org). The protocol required those with a positive result on either test to be implanted with an ICD. If both tests were negative, an ICD was recommended but the decision was left to the clinician. The mean LVEF was 28%. Preliminary data showed that no arrhythmic events were observed in 501 patients. The remaining 65 experienced VT/VF, SCD, or antitachycardia pacing for VT termination; the event rate was 7.5% at one year and 14% at two years. The PPV of both tests was about 10% or less, and their NPV were nearly the same, about 96%. The PPV of EPS remained high over two years of follow-up, but the predictive value of TWA fell off after about 12 to 15 months, suggesting a potential need for periodic TWA for risk stratification [36]. 

Chow *et al. *also evaluated the predictive value of TWA in a multicenter prospective trial of 768 patients with ischemic cardiomyopathy, LVEF ≤35% and no prior history of ventricular arrhythmia. The primary endpoint for the study was all-cause mortality and the secondary endpoint was a combination of cause-specific mortality and appropriate ICD shocks. Mean follow-up was 18±10 months. A non-negative TWA was associated with a significantly higher risk for all-cause (HR=2.24, p=0.002) and arrhythmic mortality (HR=2.29, p=0.049). In patients with ICDs, a non-negative TWA showed a trend toward higher risk for appropriate ICD shocks (HR=3.79, p=0.07). When the analysis was limited only to those patients who tested TWA positive or negative, a positive TWA was associated with higher all-cause mortality (HR=2.08, p=0.01) and a trend toward higher arrhythmic mortality (HR=2.03, p=0.13) [37]. 

**Fig. (2):**
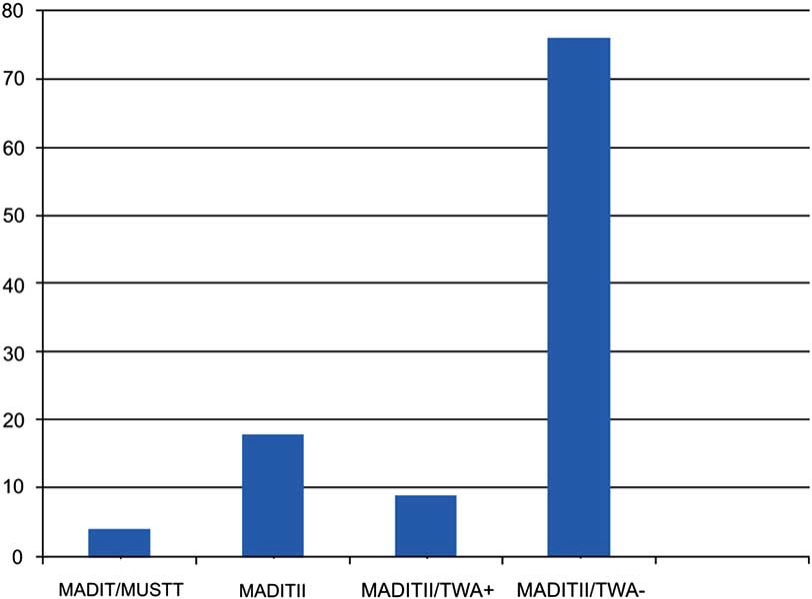
The number needed to treat to save a life varies depending on the risk stratification tools used: 4 when MADIT I/MUSTT risk stratification was used; 18 in MADIT II. However, if TWA was used in MADIT II-like patients, the number needed to treat to save a life changes to 9 if TWA non-negative or 76 if TWA negative. TWA=T wave alternans.

After multivariable adjustment, among the 392 (51%) patients who received ICDs in this multicenter trial, ICDs were associated with lower all-cause mortality in non-negative TWA patients (HR=0.45, p=0.003) but not in the negative TWA patients (HR=0.85, p=0.73), with the mortality benefit in non-negative TWA patients largely due to reduction of arrhythmic mortality (p=0.004). The NNT with an ICD for two years to save one life was 9 among non-negative TWA patients and 76 among negative TWA patients [38] (Fig. **2**). Moreover, the observed mortality in this trial was 8.26% (n=254) versus 15.17% (n=514) in the negative TWA and non-negative TWA groups, respectively. In our opinion, the mortality in both groups is clinically significant and, therefore, it cannot be concluded that there is no significant benefit from ICD therapy in the negative TWA group, since data from seven primary prevention ICD trials showed an average 28% RRR and 3% absolute risk reduction of death in ICD treated patients as compared to medical therapy. 

Recently, the MASTER I trial shed some light on this clinical dilemma. In this study, 654 patients with a MADIT II indication for ICD implantation were prospectively followed for a minimum of two years. Preliminary data analysis from 575 patients was reported at the 2007 AHA Annual Scientific Sessions (Heartwire/The Heart.org). The primary end point of life-threatening ventricular tachyarrhythmic events (as assessed by ICD shocks) was not significantly different between patients with negative and non-negative TWA (10.3% *vs*. 13.3%, p=0.37). Mortality results showed that all-cause deaths were increased in the patients with non-negative tests, but this appeared to be accounted for mainly by an increase in noncardiac deaths. The investigators concluded that TWA testing should not be used to withhold ICDs in MADIT II-indicated patients [39]. 

Similarly, TWA failed to predict SCD, sustained VT/VF, or appropriate ICD therapy (HR 1.28, p=0.46) among 490 patients with class II/III NYHA enrolled in a substudy from the Sudden Cardiac Death in Heart Failure (SCD-HeFT) trial [40]. Therefore, we can not conclusively say that patients with LVEF ≤30% do not benefit from prophylactic ICD therapy, regardless of their TWA test results. However, we can say that patients with negative TWA and LVEF ≤ 30% seem to have less benefit. This observation could be clinically relevant when patients are informed about the risks and benefits of prophylactic ICD therapy, since patients should be involved in the decision making about their care. 

The role of TWA in the risk stratification of patients with LVEF >35% has not been well studied. 

Inama *et al. *reported a significant correlation of inducibility of VT during EPS and positive or indeterminate TWA among 85 athletes with normal LVEF and complex ventricular arrhythmias [41]. 

The predictive value of TWA for overall mortality in a general population was tested in the Finnish Cardiovascular Study in 1037 consecutive patients referred for an exercise test. During a follow-up of 44±7 months, 59 patients died. Adjusted multivariate analysis demonstrated a statistically significant relative risk of SCD (RR= 7.4, p<0.001; PPV=8% and NPV=98.6%) and all-cause mortality (RR=3.3, p=0.001; PPV=14.9% and NPV=95.2%) among subjects with TWA >65 µV. LVEF was reported in 529 patients (65±15%). There were 67 patients (12.7%) with an EF <50%, and 8 patients (1.5%) presented with an EF <30%. Although SCD and mortality risk were not adjusted to LVEF, this study does suggest that TWA could add prognostive value to routine exercise stress testing in the general population [42]. 

**Fig. (3):**
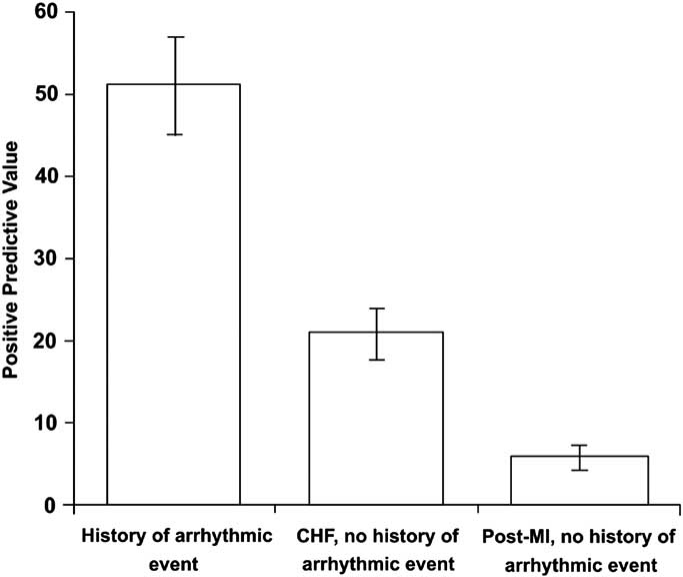
The positive predictive value of T wave alternans varies depending on the pretest probability for arrhythmic events of the population being studied. For example, the positive predictive value can vary from 25% in congestive heart failure patients to 6% in post-myocardial infarction patients. (Reprinted with permission Elsevier Limited).

However, the predictive value of TWA varies depending on the pretest probability for arrhythmic events of the population being studied. For example, the PPV varied depending on the population of patients studied; it was 25.5% at 18 months in heart failure patients and 6% in post-MI patients (p<0.0001) (Fig. **3**). Nonetheless, the NPV remained adequate in both groups, 96.3% versus 99.4%, respectively [29]. 

Future risk stratification studies will likely use a combination of risk markers, perhaps even in combination with genetic profiling, for SCD risk stratification in larger population groups that include much higher numbers of potential SCD victims. 

### Electrophysiology Studies

The predictive value of inducing sustained VT/VF during EPS (positive EPS) was tested in two large prospective multicenter trials (MADIT, MUSTT). These trials demonstrated that a positive EPS in patients with prior MI, LVEF ≤40% and NSVT identifies populations with a substantial benefit from prophylactic ICD therapy, with a 56% and 76% RRR of overall mortality and cardiac arrest and arrhythmic death, respectively [43-45]. 

More specifically, the prognostic value of positive EPS in 353 MUSTT patients who were randomized not to receive antiarrhythmic therapy was compared to that of negative EPS among 1397 patients who were followed in the MUSTT registry. The primary end point was cardiac arrest or death from arrhythmia. Patients were followed for a median of 39 months. The two-year and five-year rates of cardiac arrest or death due to arrhythmia were 12 and 24 %, respectively, among the patients in the registry, as compared with 18 and 32% among the patients with inducible tachyarrhythmias who were assigned to no antiarrhythmic therapy (adjusted P<0.001). Overall mortality after five years was 48% among the patients with inducible tachyarrhythmias, as compared with 44% among the patients in the registry (adjusted P=0.005) [46]. However, even patients without inducible tachyarrhythmias had a relatively high risk of death, indicating that patients with negative EPS still had high mortality rates and could potentially benefit from prophylactic ICD therapy. 

The presence of inducible sustained  ventricular tachycardia proved to be a relatively specific predictor of death from arrhythmia in this group of patients. The proportion of deaths classified as due to arrhythmia was greater among the patients with inducible tachyarrhythmias who were randomly assigned to no antiarrhythmic therapy than among the patients in the registry, in whom sustained tachyarrhythmias could not be induced. In the MUSTT registry, the two year negative predictive value of EPS for cardiac arrest or death due to arrhythmia was 88%. However, the negative predictive value of EPS may diminish with time as coronary artery disease and LV dysfunction worsen [46]. 

As mentioned earlier, data analysis from 674 patients enrolled in the MUSTT study who did not receive antiarrhythmic or ICD therapy revealed that the prognostic value of LVEF and inducible sustained ventricular tachycardia is influenced by other variables, such as functional class, history of heart failure, NSVT, age, left ventricular conduction abnormalities, and atrial fibrillation [19]. These findings suggest that risk stratification algorithms should include several markers of risk, especially in patients with LVEF >35%. 

In the MADIT II study, 593 (82%) of 720 patients randomized to the ICD arm also underwent EPS. Inducible patients had a greater likelihood of experiencing ICD therapy for spontaneous VT than non-inducible patients (p=0.023). However, ICD therapy for spontaneous VF was less frequent in inducible patients. Therefore, the two-year event rate for combined VT/VF was 29.4% for inducible patients and 25.5% for noninducible patients. (p = 0.280). In other words, a positive EPS is a good predictor of VT, but not a good predictor of VF [47]. 

These data reinforce the concept that EPS provides important but limited prognostic information. Today, EPS still has a role in the risk stratification of asymptomatic patients with NSVT, coronary artery disease and LVEF between 30-40% in the absence of heart failure symptoms. It also has a significant role in the risk stratification of symptomatic patients (syncope) and LVEF >40%. 

### Signal Averaged Electrocardiography

The SAECG is a highly amplified and signal-processed ECG that can detect microvolt-level electrical potentials in the terminal QRS complex, known as late potentials. These arise from scarred myocardium, which can be a source of reentrant malignant ventricular arrhythmias. An abnormal SAECG (filtered QRS duration >114 ms) has been correlated with inducibility of VT [48]. 

A meta-analysis of 20 clinical studies in post-MI patients (n=9883) showed that an abnormal SAECG had a positive predictive value ranging from 6 to 35% in forcasting major arrhythmic events [49]. 

The predictive value of SAECG was evaluated in 1268 patients (66%) out of 1925 patients enrolled in the MUSTT trial who had a baseline SAECG. In subjects with an abnormal SAECG, the 5-year rates of the primary end point of arrhythmic death or cardiac arrest (28% *vs.* 17%, *P*=0.0001), cardiac death (37% *vs.* 25%, *P*=0.0001), and total mortality (43% *vs.* 35%, *P*=0.0001) were significantly higher [48]. 

In the CABG Patch Trial, 900 patients scheduled for elective coronary bypass surgery with an LVEF ≤35% and an abnormal SAECG were randomly assigned to therapy with an ICD (446 patients) or to the control group (454 patients). The primary endpoint of the study was overall mortality. During an average follow-up of 32±16 months, there were 101 deaths in the defibrillator group and 95 in the control group (hazard ratio =1.07; P=0.64). Revascularization may have altered the immediate prognosis irrespective of the SAECG and EF. On the other hand, in many patients, EF may have improved after revascularization, or the substrate for arrhythmogenesis could have been substantially altered. Further analysis of the CABG Patch study showed that ICD use reduced arrhythmic death by 45%, although total mortality was not reduced because of an increase in non-arrhythmic deaths [50]. 

Observations from these studies are not necessarily contradictory, since they compared different patient populations and different definitions of an abnormal SAECG were used (filtered QRS duration >114 ms in MUSTT *vs*. filtered QRS duration >114 ms; root-mean-square voltage in the terminal 40 msec of the QRS complex <20 µV; or duration of the terminal filtered QRS complex at <40 µV >38 ms in the CABG Patch Trial). Hence, the SAECG might still have a role in risk stratification for arrhythmic death. 

## MARKERS OF ABNORMAL AUTONOMIC BALANCE

There is a close relation between the autonomic nervous system and arrhythmogenesis. Heart rate variability (HRV) and baroreflex sensitivity (BRS) have become important methods for assessing cardiovascular autonomic regulation and have been extensively studied for their value as predictors of total mortality, SCD, and the occurrence of ventricular tachyarrhythmias. Initial animal experiments in dogs with healed myocardial infarction by Schwartz *et al. *demonstrated that the presence of reduced BRS is associated with a greater susceptibility to ventricular fibrillation during subsequent ischemic episodes [20,51-53]. 

A meta-analysis of 11 clinical studies in post-MI patients (n=5719) showed that an abnormal HRV had a PPV ranging from 9 to 54% in predicting major arrhythmic events (49). On the other hand, in a single center, prospective study of 700 post MI patients on adequate beta blocker therapy, Huikuri *et al. *reported that autonomic markers (HVR, BRS) did not predict SCD [23]. 

In the ATRAMI trial, the prognostic value of HRV and BRS were assessed prospectively in 1284 post-MI patients. Low HRV (Standard deviation of R-R interval, SDNN <70 ms) or BRS (<3.0 ms per mm Hg) values carried a significant mortality risk (HR=3.2 and 2.8, respectively). The association of low SDNN and BRS further increased risk; the 2-year mortality was 17% when both were below the cut-offs and 2% (p<0.0001) when both were well preserved (SDNN >105 ms, BRS >6.1 ms per mm Hg). The association of low SDNN or BRS with LVEF <35% carried a relative risk of 6.7 or 8.7 (4.3-17.6), respectively, compared with patients with LVEF ≥35% and less compromised SDNN (≥70 ms) and BRS (≥3 ms per mm Hg). These results suggest that assessment of autonomic balance provides important prognostic information in post MI patients [54]. 

In the DINAMIT trial, 674 patients with a recent MI (6-40 days), LVEF ≤ 35% and low HRV (SDNN <70ms) or a mean RR interval ≤ 750 ms were randomly assigned to the ICD group (n=332) or to the control group (n=342). The primary outcome was mortality from any cause. During a mean follow-up period of 30±13 months, there was no difference in overall mortality between the two treatment groups. A significant decrease in arrhythmic death was observed, 12 (ICD group) versus 29 (control group) (p=0.009). However, this effect was offset by a significant increase in non-arrhythmic death, 50 (ICD group) *vs.* 29 (control group), p=0.02. The reason for this increase in non-arrhythmic mortality in the ICD group is not clear, since it has not been observed in other primary prevention ICD trials. The predictive value of abnormal HRV (SDNN <60 ms) was not clinically significant [55]. 

## CONCLUSION

We have made significant progress in the risk stratification of SCD. The combination of various risk markers in primary prevention ICD trials (LVEF, NSVT, EPS) have allowed the identification of patients at risk who could benefit from prophylactic ICD therapy. At the present time, LVEF remains the single most important marker for risk stratification. According to current guidelines, most patients with LVEF <35% could benefit from prophylactic ICD implantation, particularly in the setting of symptomatic heart failure [56]. Other risk stratifiers, such as T wave alternans, give additional prognostic information. However, because there is no method with a negative predictive value of 100%, most physicians have been reluctant to base decisions on ICD implantation solely on the basis of such test results. On the other hand, risk markers can been useful in borderline cases or when patients or physicians are reluctant to pursue ICD implantation without further data. 

In addition, current risk stratification strategies fail to identify patients at risk of SCD in larger population groups encompassing a greater number of potential SCD victims [11]. In other words, current guidelines for prophylactic ICD therapy only identify a minority of the SCD victims. 

It is clear that patients with normal or mildly depressed LVEFs (>35%) have a better prognosis and lower risk of serious cardiac arrhythmias. We have shown from small and large, single center and multicenter clinical trials that markers of abnormal substrate or structural heart disease (LVEF, NSVT, frequent ventricular ectopy, QRS duration), markers of abnormal repolarization or electrical instability (EPS, TWA, SAECG) and markers of abnormal autonomic balance (HRV and BRS) provide important prognostic information in a wide range of clinical situations. The challenge that lays ahead is the implementation of risk stratification strategies that can reliably predict risk in patients with LVEF >35%. We have also shown that in this group of patients, risk stratification strategies will require the use of a combination of several risk markers, but risk stratification strategies will need to be tested in large prospective multicenter clinical trials before they can be incorporated into daily clinical practice. 

## ABBREVIATIONS

ARR: = Absolute risk reduction
BRS: = Baroreceptor sensitivity
EPS: = Electrophysiology study
HR: = Hazard ratio
HRV: = Heart rate variability
ICD: = Implantable cardioverter defibrillator
LVEF: = Left ventricular ejection fraction
MI: = Myocardial infarction
NNT: = Number needed to treat
NPV: = Negative predictive value
NSVT: = Non-sustained ventricular tachycardia
PPV: = Positive predictive value
RRR: = Relative risk reduction
SAECG: = Signal averaged electrocardiography
SCD: = Sudden cardiac death
SDNN: = Standard deviation of R-R interval
TWA: = T wave alternans
